# Draft Genome Sequence of Pectobacterium atrosepticum PB72 and Complete Genome Sequence of the Specific Bacteriophage PP90

**DOI:** 10.1128/genomeA.00473-18

**Published:** 2018-07-05

**Authors:** Mikhail M. Shneider, Anastasia P. Kabanova, Aleksei A. Korzhenkov, Kirill K. Miroshnikov, Ngoc Ha Vo Thi, Stepan V. Toshchakov, Konstantin A. Miroshnikov, Alexander N. Ignatov

**Affiliations:** a Shemyakin & Ovchinnikov Institute of Bioorganic Chemistry RAS, Moscow, Russia; b PhytoEngineering R&D Center, LLC, Moscow Region, Russia; c Immanuel Kant Baltic Federal University, Kaliningrad, Russia; d Winogradsky Institute of Microbiology, Research Center of Biotechnology RAS, Moscow, Russia; e Peoples’ Friendship University of Russia, Moscow, Russia

## Abstract

We present the draft genome sequence of Pectobacterium atrosepticum strain PB72 infecting potatoes in Russia. PB72 is similar to the previously reported strain 21A. Considering potential biocontrol of this pathogen, an infectious bacteriophage was isolated and characterized. Phage vB_PatP_PP90 is a lytic podovirus of narrow host range belonging to the *KP34virus* genus.

## Abstract (precis)

We present the draft genome sequence of Pectobacterium atrosepticum strain PB72 infecting potatoes in Russia. PB72 is similar to the previously reported strain 21A.

## GENOME ANNOUNCEMENT

Pectobacterium atrosepticum is a plant pathogen (1) associated with blackleg disease of potatoes (2, 3), with sequenced strain SCRI1043 (4) used as a model (5). P. atrosepticum is rated among the most destructive plant pathogens in Russia, and bacteriophage application is used as a biocontrol method (6, 7).

Both P. atrosepticum strain PB72 and phage PP90 were isolated from diseased potatoes in the Moscow Region of Russia in 2014. Bacteria were grown in LB medium at 27°C, and the phage was propagated using strain PB72 as a host. Bacterial and phage genomic DNA was extracted using a standard phenol-chloroform protocol and subjected to ultrasound fragmentation by a Bioruptor (Diagenode) to obtain a mean fragment size of 500 bp. Fragment libraries were constructed using a NEBNext Ultra kit (New England Biolabs). Sequencing was performed on an Illumina MiSeq platform using paired-end 150-bp reads. After sequencing, all reads were subjected to stringent quality filtering and trimming with CLC Genomics Workbench 10.0 (Qiagen). Sequencing adapters were trimmed with the SeqPrep tool (https://github.com/jstjohn/SeqPrep). Reads of both PB72 and PP90 were assembled with SPAdes 3.10.0 (8).

A total of 1,133,659 read pairs were used for *de novo* assembly of strain PB72. The obtained draft genomic assembly consisted of 50 scaffolds of 4,986,032 nucleotides (nt) in total and an *N*_50_ value of 238,550 nt, with average read coverage of 67×. Genome annotation was performed using Prokka (9). Coding sequences were predicted using Prodigal (10), tRNA genes and transfer-messenger RNA were predicted by ARAGORN (11), rRNA genes by Barrnap (http://www.vicbioinformatics.com/software.barrnap.shtml), and noncoding RNAs by Infernal (12). CRISPRs were detected by MinCED (https://github.com/ctSkennerton/minced). The PB72 genome with a GC content of 51.1% contains 4,421 protein coding sequences, 10 rRNA genes, 70 tRNAs, and 2 CRISPR loci. Organization of the PB72 chromosome and gene content and order are very similar to those of P. atrosepticum 21A (13), except for mostly phage-related horizontally transferred sequences, accounting for 35 unique genes in PB72. No plasmids were identified among the reads, in contrast to strain 21A.

Bacteriophage PP90 has a very narrow host range, not infecting P. atrosepticum 21A and SCRI1043 or 60 other tested Pectobacterium and Dickeya strains. It forms 1- to 2-mm plaques with a pronounced halo. Negative staining electron microscopy shows podoviral phage morphology, and thus the phage can be referred to as vB_PatP_PP90 (14).

The genome of PP90 consists of 44,570 bp with a GC content of 56%. Average genome coverage was 64×. Genome annotation using GeneMarkS (15), Glimmer (16), RAST (17), and BLASTP (18) reveals 56 ORFs and no tRNAs. The closest (95.61% average nucleotide identity [ANI]) published phage isolate is P. atrosepticum phage Peat1 (NC_029081) (19), but PP90 has a unique orf11, orf14, and orf16. The general genome layout and the composition of the lysis module make PP90 a member of the genus *KP34virus*. To date, all characterized P. atrosepticum bacteriophages were isolated using strain SCRI1043 or uncharacterized strains (20, 21). Hence, this work is a first report of the phage infecting a 21A-group strain that is genetically diverse from SCRI1043 (13, 22).

### Accession number(s).

The NCBI nucleotide sequence accession numbers for this project are PDDK00000000 for the P. atrosepticum PB72 genome assembly and KX278419 for bacteriophage PP90.
